# Case report: microcephaly associated with Zika virus infection, Colombia

**DOI:** 10.1186/s12879-017-2522-6

**Published:** 2017-06-13

**Authors:** Salim Mattar, Carolina Ojeda, Janna Arboleda, German Arrieta, Irene Bosch, Ingrid Botia, Nelson Alvis-Guzman, Carlos Perez-Yepes, Lee Gerhke, German Montero

**Affiliations:** 1 Universidad de Cordoba, Instituto de Investigaciones Biologicas del Tropico, Monteria, Colombia; 2 Clinic Salud Social, Sincelejo, Colombia; 3 CECAR, Corporacion Universitaria del Caribe, Public health group, Sincelejo, Colombia; 4 MIT, Cambridge, MA USA; 5 Universidad de Cartagena, Health Economics group, Cartagena, Colombia; 6 Clinica IMAT, Monteria, Colombia

**Keywords:** Zika, Microcephaly, Colombia, Central nervous system vascular malformations, Pediatrics

## Abstract

**Background:**

Recently there has been a large outbreak of Zika virus infections in Colombia, South America. The epidemic began in September 2015 and continued to April 2017, for the total number of Zika cases reported of 107,870. For those confirmed Zika cases, there were nearly 20,000 (18.5%) suspected to be pregnant women, resulting in 157 confirmed cases of microcephaly in newborns reported by their health government agency. There is a clear under-estimation of the total number of cases and in addition no prior publications have been published to demonstrate the clinical aspects of the Zika infection in Colombia. We characterized one Zika presentation to be able to compare and contrast with other cases of Zika infection already reported in the literature.

**Case presentation:**

In this case report, we demonstrate congenital microcephaly at week 19 of gestation in a 34-year-old mother who showed symptoms compatible with Zika virus infection from Sincelejo, State of Sucre, in the Colombian Caribbean. Zika virus RNA was detected in the placenta using real-time reverse transcriptase polymerase chain reaction (RT-PCR). At week 25, the fetus weigh estimate was 770 g, had a cephalic perimeter of 20.2 cm (5th percentile), ventriculomegaly on the right side and dilatation of the fourth ventricle. At week 32, the microcephaly was confirmed with a cephalic perimeter of 22 cm, dilatation of the posterior atrium to 13 mm, an abnormally small cerebellum (29 mm), and an augmented cisterna magna. At birth (39 weeks by cesarean section), the head circumference was 27.5 cm, and computerized axial tomography (Siemens Corp, 32-slides) confirmed microcephaly with calcifications.

**Conclusion:**

We report a first case of maternal Zika virus infection associated with fetal microcephaly in Colombia and confirmed similar presentation to those observed previous in Brazil, 2015–2016.

## Background

Zika, Chikungunya and dengue viruses are tropical vector borne viral diseases that are all endemic in Colombia, South America. During the last 16 months, Colombia has experienced a large outbreak in Zika infection. From September 2015 through May 20th 2017, 107,870 reported cases. However, only 9.5% of these were laboratory confirmed at the Colombian National Institute of Health (INS) [1, 2]. Infection with Zika virus during pregnancy has been linked to the occurrence of microcephaly via transplacental infection of the developing fetus and subsequent neuronal cell death. In South America, the Zika virus outbreak began in Brazil in May 2015, and in Colombia local transmission, began about 5 months later, in October 2015. The disease has spread to the entire country where *Aedes aegypti* is as well established vector. Similar to other countries in the Americas affected by Zika virus, the increases of congenital syndromes including microcephaly cases and Guillan Barre syndrome appeared abruptly coincident with the Zika virus disease (ZVD) outbreak. In 20 May 2017, there was a calculation of 0.22% of Colombian population that has been affected by ZVD including almost 20,000 cases of infected pregnant women [2]. This is an alarming situation because of congenital defects associated with ZIKV infection. Such congenital defects cases have increased since 2016 [1–3]. The number of cases confirmed in laboratories is lower (9.7%) than reported in other countries [3, 4]. Most cases confirmed in laboratories correspond to those notified in pregnant women (64.95%). Currently, the Instituto Nacional de Salud of Colombia (INS) continues active surveillance for birth defects, including microcephaly [2–4]. Up to date, in Colombia, there is no published report of microcephaly associated with Zika virus infection. We report here a case of microcephaly in a child exposed in utero via maternal infection with Zika virus.

## Case presentation

We describe a case of microcephaly associated with Zika virus infection in an infant born on 17 July 2016. The 34-year-old mother showed symptoms compatible with Zika infection: rash at week 19 of gestation, living in an endemic area during the last 5 years and with no immunodeficiency or autoimmune diseases. Laboratory tests showed no evidence of diabetes or blood-pressure-related disorders. In her medical history, the mother indicated no drug or alcohol use or smoking during pregnancy. No active infection against toxoplasma, rubella, cytomegalovirus, herpes, syphilis, Chikungunya, dengue, and human immune deficiency virus (HIV) was detected in the mother’s serum as per Hospital laboratory testing report.

**Fig. 1:**
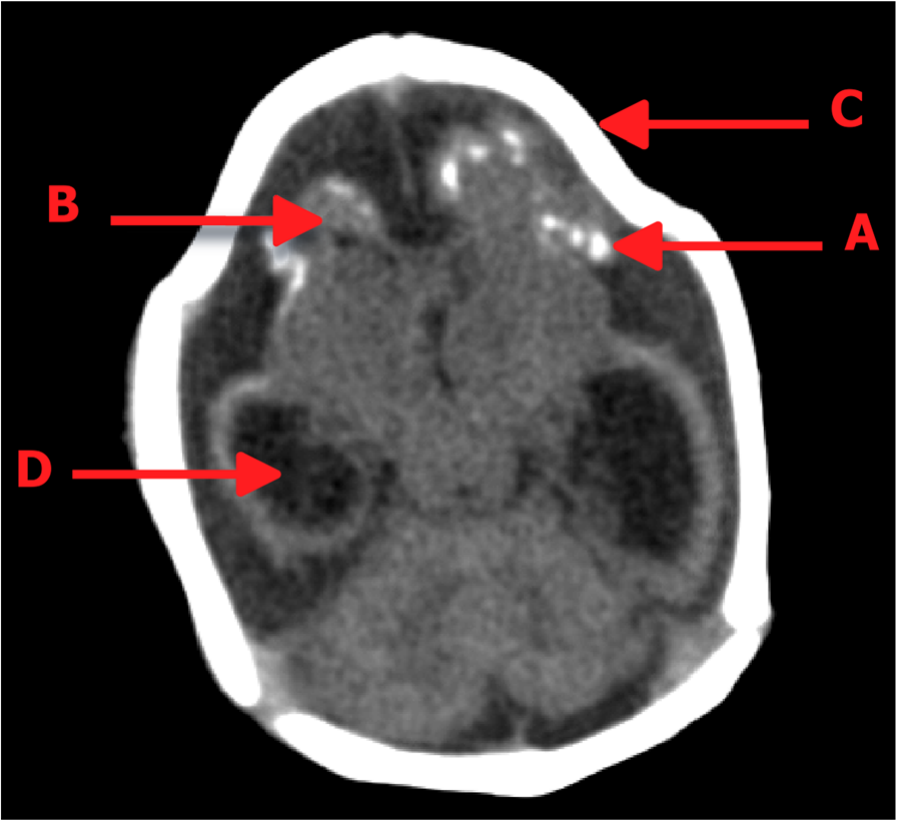
**a** Intracebral calcifications of the congenital prenatal infectious type. **b** Cerebral hypoplasia. **c** Microcephaly. **d** Supratentorial hydrocephalus. CAT was carried out using axial cuts from the base to convexity. CAT scan shows: brain size diminished with deformity with evidence of congenital microcephaly. Cerebral parenchyme volume is diminished due to hypoplasia associated to a ventricular dilated suparatentorial. Amplitude of the furrows and intraparenchymal cortical calcifications by malformation and subarachnoid spaces, there is no displacement of midline structures, brain stem and cerebellum are preserved

At 16 weeks, ultrasound showed a head circumference of 11.5 cm and fetal weight estimate of 219 g. At 25 weeks, the fetus weight was 770 g, with a cephalic perimeter of 20.2 cm (5th percentile), ventriculomegaly on the right side and dilatation of the fourth ventricle. A third ultrasound at week 32 showed a cephalic perimeter of 22 cm and confirmed the microcephaly diagnosis with dilatation of the posterior atrium to 13 mm, an abnormally small cerebellum (29 mm), and an augmented cisterna magna. At birth, the anterior fontanelle was closed; the facies were symmetrical; normal ocular apertures; normal spleen and urinary size determined via ultrasound; good respiratory pattern; normal palate; normal heart rate with no alterations and no megalia detected. At birth (39 weeks by cesarean section), the infant’s head circumference was 27.5 cm and the computerized axial tomography scan confirmed microcephaly with calcifications (Fig. 1).

Zika virus RNA was extracted using standard procedures from the placenta, Zika genome was detected in the placenta using real-time reverse transcriptase polymerase chain reaction (RT-PCR) according to Zika specific PCR previously published [4–6]. Detection of Zika virus in umbilical cord and serum of the neonate via RT-PCR were unsuccessful. We utilized the commercial Zika virus test (Biocan, Canada). IgM antibodies to Zika were detected in the serum of the mother. Currently, the newborn is 9 months old with strabismus, weak muscle tone in his neck, recurrent neurological cry, repeated respiratory infections, neurological impairment and possibly normal hearing (data no published).

## Discussion

We report a first case in Colombia of fetal Zika virus infection via maternal transmission resulting in microcephaly. It is important to note that during the previous five years the mother lived in Sincelejo, Sucre State, an endemic area for Zika in the Colombian Caribbean. As reported by the INS, Zika virus has presumably infected more than 1655 cases in Sucre State, representing 1.53% of the total Colombian population, this data would confirm the high prevalence of Zika in the area where the clinical case derives from. Since the Zika outbreak began in Colombia neurologic anomalies including microcephaly 157 accumulated total cases have been reported to the INS [1, 2]. The data illustrate the large epidemiologic impact of Zika virus infection on the population of Sucre state. In the present report, Zika viral RNA was detected in the placenta by RT-PCR and IgM antibodies to Zika were detected in the mother’s serum. It is not known exactly how Zika virus reaches the embryo, but it remains in the placenta until delivery and presumably crosses to the fetus from the placenta [6]. However, there are reports of RNA being found in symptomatic pregnant women up to 53 days after initial symptoms [6].

During pregnancy, infection with Zika virus has been associated with microcephaly and other fetal brain abnormalities [7–10]. The first ultrasound detection of fetal brain abnormalities associated with Zika virus varies from between 20 and 40 weeks of gestation [3, 7, 8]. In the present study abnormalities were confirmed at 25 weeks. In Brazil investigators, observed abnormalities at early as 21 weeks of gestation [3, 9]. Although microcephaly was confirmed by ultrasonography at week 25, the mother decided not to interrupt her pregnancy. This decision was likely related to religious and cultural beliefs of many Colombian women who would prefer to completion of the pregnancy, Brazilian investigators have established a range 8–35 (mean = 27.8) weeks of gestation as being critical for Zika related microcephaly, demonstrating the criticality of infection during the 1st and 2nd trimesters for fetal brain abnormalities to be observed [8]. The diagnosis of our case at week 25 of gestation is in agreement with the Brazilian findings [8]. Further, we have observed two newborn babies affected with Dandy Walker syndrome associated with Zika virus infection (data no published).

Between 2010 and 2017, neurologic complications (microcephaly) in French Polynesia Brazil and Colombia have increased dramatically [4, 5, 9, 10]. These brain malformations in those countries coincided with Zika virus outbreaks [10–12]. The relationship between Zika virus and microcephaly was first suspected in Brazil in 2015. Based on a retrospective study in French Polynesia and the new emerging public health problem in Brazil, the Pan American Health Organization declared an alert on 17 November 2015 [7]. In January 2016, Brazil reported 3893 cases of microcephaly. In February 2016, the World Health Organization declared a global health emergency [7]. In the Colombian public health system, declaration of microcephaly cases was not obligatory but the currently identified cases Zika-associated microcephaly confirm the need for increased vigilance and monitoring. In Colombia, before the current thrush of microcephaly cases appeared, an etiologic relationship between Zika virus and microcephaly was not suspected and a relationship between Zika virus and central nervous system anomalies was doubted.

The 19,935 cases of pregnant women with clinically compatible Zika virus infection suggest that Colombia may experience a dramatic increase in the number of microcephaly cases. Out of a total of 19,935 pregnant women, 157 (0.79%) cases of congenital microcephaly associated with Zika virus infection have been reported; it is unknown whether this number will increase in the near future. It is also unknown the reasons why some pregnant women infected by Zika virus give birth to healthy babies and others do not. Individual genetic background or modulation of the immune system can perhaps be involved in the pathways for the virus to reach trophoblasts. In Colombia, before microcephaly cases appeared, an etiologic relationship between Zika virus and microcephaly was not demonstrated and a relationship between Zika virus and central nervous system anomalies was doubted.

In the Colombian Caribbean, we have witnessed an increase in microcephaly associated with Zika virus prevalence and exposure during pregnancy [2–4]. Some of the exposed mothers will be delivering their babies during the next four months, and our current research suggests that the number of fetal microcephaly cases will increase. Unfortunately, it is too soon to establish the exact incidence of microcephaly in the already pregnant women in Colombia. Brazilian officials have detected fetal abnormalities in 12/42 (29%) of Zika virus-positive women, but we do not know whether this rate will be similar in Colombia [4, 7, 9]. The cost of treatment to improve neurodevelopmental deficiencies in children due to Zika mediated neuronal cell death remains a major concern for Colombia. Early and low-cost serodiagnosis of Zika virus infection will be critical to avoid high degrees of unseen consequences to viral infection. Serologic detection of Zika is problematic in Colombia and Latin-American due to cross virus reactivity in current imuno-assays. Besides the ordinary screening tests (toxoplasma, rubella, cytomegalovirus, herpes, syphilis and HIV) the Colombian Minister of Health must quickly ensure that pregnant women be aware of and be protected against new agents such Zika, also, better characterized viruses like Chikungunya and Dengue must be included in any viral screening that for the past or present invasion of a viral mediated congenital defect such as microcephaly. All inborn errors of cell function present a significant weight on current drug development, with many effective therapies being too expensive to be developed. We have significant economic progress to make ensure that the impact of our public health measure be maximized.

## Conclusions

Here, we report the for the first time a case in Colombia, South America of fetal microcephaly associated with maternal Zika virus infection. There have been 19,935 suspected cases of Zika infection of pregnant women in Colombia and these numbers must with a relatively small number (*n* = 157) associated with fetal microcephaly (0.79%). Zika virus related microcephaly needs to be monitored closely in those countries experiencing outbreaks so as to provide accurate information on risk to the unborn fetus. The experience in Colombia as well as other countries provides an important epidemiologic example to predict risk for other countries. This and other reports provide critical information for world medical and community public health.

## Abbreviations

CAT: Computer axial tomography
GBS: Guillain-Barre syndrome
HIV: Human immune deficiency virus
INS: Colombian National Institute of Health
RT-PCR: Real-time reverse transcription polymerase chain reaction
ZVD: Zika virus disease
